# Paralysie obstétricale bilatérale du plexus brachial

**DOI:** 10.11604/pamj.2014.18.184.4866

**Published:** 2014-07-03

**Authors:** Wassia Kessomtini, Wafa Chebbi

**Affiliations:** 1 Unité de Médecine Physique, CHU Taher Sfar Mahdia, 5100 Mahdia, Tunisie; 2 Service de Médecine Interne, CHU Taher Sfar Mahdia, 5100 Mahdia, Tunisie

**Keywords:** Paralysie obstétricale, plexus brachial, bilatérale, obstetric paralysis, brachial plexus, bilateral

## Image en médecine

La paralysie obstétricale du plexus brachial est la plus fréquente des traumatismes obstétricaux. Elle reste une complication obstétricale redoutée qui doit faire l'objet d'un suivi particulier car elle engage le pronostic fonctionnel du membre. Sa fréquence varie de 0,42 à 5,1 cas pour 1000 naissances vivantes. La paralysie étant unilatérale, le diagnostic est souvent évident à la naissance après un accouchement laborieux. Le membre supérieur présente une paralysie flasque. Il est hypotonique et ballant en contraste avec l'hypertonie en flexion physiologique du côté normal. Les formes bilatérales de la paralysie obstétricale du plexus brachial sont exceptionnelles (1%) et s'observe presque exclusivement dans la présentation de siège. Le traitement repose sur la kinésithérapie et la chirurgie réparatrice nerveuse dans certains cas. Nous rapportons un cas rare de paralysie bilatérale obstétricale du plexus brachial. Il s'agissait d'un nourrisson âgé de 3 mois, issu d'un accouchement présentation de siège, adressé à l'unité de médecine physique pour un déficit moteur des deux membres supérieurs. L'examen montrait une paralysie du plexus brachial bilatérale type C5 C6 sans signes de gravité associés. Le reflexe de moro était pathologique des deux cotés ainsi que la manœuvre de Foulard. Après 3 mois de rééducation à raison de 3 séances par semaine, aucune récupération n'est observée au niveau du deltoïde ni au niveau du biceps brachial. L’électromyogramme confirmait la dénervation bilatérale au niveau de C5, C6 et C7. L'IRM du plexus brachial révélait des pseudo-méningocèles bilatérales des racines C5, C6 et C7, témoignant d'une section nerveuse bilatérale. Le nourrisson était adressé pour chirurgie nerveuse.

**Figure 1:**
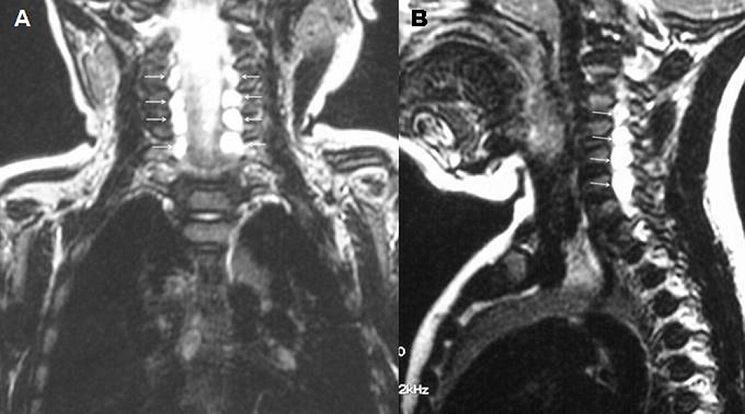
IRM des plexus brachiaux. Séquences Fiesta: A) dans le plan coronal et B) dans le plan sagittal; Pseudo- méningocèles bilatérales des racines C5, C6 et C7 (flèches blanches)

